# From trials to the public health: pre-exposure prophylaxis for HIV prevention

**Published:** 2013-03-30

**Authors:** Esper Georges Kallas, Luiz João Miraglia

**Affiliations:** a Division of Clinical Immunology and Allergy, School of Medicine, University of São Paulo, E-mail: esper.kallas@usp.br

2.2 million adults were newly HIV-infected in 2011, underscoring the urgent need for new, effective ways to prevent incident infections. ^1^ Recently, the field of HIV prevention has gathered positive results from different strategies, among different populations, and with varying effect sizes, including the treatment of HIV-positive women and men in discordant couples,^2^ ^-^ ^4^ male circumcision of HIV-negative men in sub-Saharan Africa,^5^ ^-^ ^7^ a HIV vaccine evaluated in a community-based trial among HIV-negative men and women in Thailand,^8^ the use of vaginal gel formulation of TDF for HIV prevention in women in South Africa,^9^ pre-exposure prophylaxis (PrEP) with tenofovir disoproxil fumarate (TDF) or emtricitabine and TDF (TDF-FTC) among HIV-1-serodiscordant heterosexual couples from Kenya and Uganda,^10^ and PrEP with TDF-FTC among heterosexual men and women in Africa.^11^ ^,^ ^10^ Of these interventions, PrEP is an attractive policy because it does not directly interferes with the sexual intercourse, providing people a choice on HIV prevention regardless of cultural, religious, or social harnesses.

The use of PrEP is particularly interesting in high-risk populations that have been difficult to reach with traditional prevention strategies. For example, Grant et al. have published results from the Preexposure Prophylaxis Initiative (Iniciativa Preexposicion - iPrEx) study, showing the efficacy of PrEP with a daily pill of TDFFTC among HIV-seronegative men and transgender women who have sex with men.^12^ This randomized, multinational, placebocontrolled, double-blind trial included 2499 participants in clinical sites at North and South America, South Africa, and Thailand. Of the 100 incident HIV infections, 64 occurred among the group receiving placebo, and 36 among the group receiving FTC-TDF, resulting in an estimated efficacy of 44% (95% confidence interval: 15 to 63%). The protection reached 73% in those reporting at least 90% adherence to the pill use and, in recent follow-up results, up to 99% in those with detectable drug levels in the blood in oncea-day pill regime.^13^

Although having its efficacy demonstrated, followed by the approval from the FDA,^14^ and an interim guidance published by the CDC,^15^ several concerns have been raised regarding the scalability, feasibility, and acceptability of PrEP with oral TDF-FTC.

Safety concerns have been related to renal toxicity and to the reduction in bone density. While elevated creatinine levels were not significantly different between participants receiving placebo or TDF-FTC,^11^ ^,^ ^13^ ^,^ ^16^ the latter group was found to present a clinically not relevant, but statistically significant decline in bone mineral density.^11^ ^,^ ^17^ Additional studies with longer follow-up periods will be needed to further evaluate these safety concerns and their impact on the use of PrEP.

In addition, it is possible that HIV acquisition during the use of PrEP could lead to viral resistance, and in fact five cases of viral resistance were documented in three trials published,^11^ ^-^ ^12^ although they occurred in subjects infected at enrollment, during the "window period" of HIV infection, i.e., with detectable HIV-RNA while being anti-HIV antibody negative. A trial that failed to demonstrate the efficacy of PrPEP among 1062 women receiving TDF-FTC, documented four cases of resistance to FTC, and reported low rates of adherence to the study medication evaluated through plasma drug concentration^17^.

Low adherence was also found by the iPrEx study, which will likely to be a major challenge for the implementation of PrEP, and is of central importance since improved adherence has been associated with greater efficacy.^14^ ^,^ ^16^ Approaches to support product use in the iPrEx study, and in the iPrEx Open Label Extension study (iPrEx-OLE), could help to improve adherence to PrEP outside the clinical trial setting.^17^ It is conceivable that the reported efficacy of PrEP could serve as a strong stimulus for higher pill use, in a scenario where success begets success.

Another concern is risk compensation related to PrEP use,^18^ ^,^ ^19^ although data obtained in large trials have shown the opposite: the use of a prevention pill was associated with a significant decrease of high risk sexual behavior. In the iPrEx trial, data obtained by computer assisted structure interviews demonstrated that during the 12 weeks before the interview was administered, median number of sex partners decreased from 7 to 2 and the percentage reporting unprotected receptive anal intercourses dropped from 60% to 30%.^20^ Preliminary data from the iPrEx extension have confirmed the lack of risk compensation (manuscript in preparation). Although further PrEP demonstration studies still need to confirm these observations, the use of a preventive pill tends to be synergistic to other HIV prevention strategies, as it helps bringing highly vulnerable groups to medical care and serves as a daily reminder for the importance of sexually transmitted disease prevention. Whether the durability of adherence will be limited by fatigue or enhanced by habit creation remains to be determined.

In its recent publication "The Strategic Use of Anti-retrovirals", the World Health Organization argues that difficult decisions will have to be made given the increasing range of prevention and treatment options, and that using ARVs most strategically requires careful decision-making.^1^ WHO is supporting a HIV treatment as prevention (TasP) strategy based on an incremental expansion of ARV provision among HIV-positive persons, and consider the use of PrEP as a "niche" intervention which, in certain circumstances, might complement the early initiation of ART.^1^ ^,^ ^21^ However, the WHO has also indicated the superiority of combination of various prevention strategies including non-ARV based interventions (biomedical, behavioral and/or structural ones) plus the use of ARVs.

For certain individuals and in particular circumstances, using ARVs for PrEP to prevent HIV acquisition should be strongly considered as part of such a combination. Discordant couples, sex workers, and men and transgender women who have sex with men are still highly vulnerable and the current available HIV prevention interventions have been unable to block transmission at desirable levels. As recently pointed out, men and transgender women who have sex with men are still being hardly hit by HIV/AIDS epidemic^22^ and could considerably benefit to additional prevention options such as PrEP.

The results of the large PrEP clinical trial represent a remarkable advance in the field of HIV prevention. Demonstration studies are warranted as the last step to evaluate PrEP before implementing as a public health policy. If confirmed feasible in such studies, PrEP will maximize the prevention benefits, and could be used as part of a more comprehensive strategy.
